# Acceptability and challenges of community-based rehabilitation (CBR) of leprosy patients with grade-2 disability in Bangladesh

**DOI:** 10.1371/journal.pgph.0006994

**Published:** 2026-08-07

**Authors:** Khursheda Akhtar, Fahmida Khanam, Amena Khatun Mita, Most. Sadika Rahman, Tasnim Shahriar Osama, Md Abdullah Saeed Khan

**Affiliations:** 1 Department of Public Health and Hospital Administration, National Institute of Preventive and Social Medicine, Dhaka, Bangladesh; 2 Department of Microbiology, National Institute of Preventive and Social Medicine, Dhaka, Bangladesh; 3 Department of Dental Public Health, Islami Bank Medical College and Hospital, Rajshahi, Bangladesh; 4 Department of Community Medicine, National Institute of Preventive and Social Medicine, Dhaka, Bangladesh; 5 Department of Biomedical Engineering, National Institute of Preventive and Social Medicine, Dhaka, Bangladesh; PLOS: Public Library of Science, UNITED STATES OF AMERICA

## Abstract

Leprosy patients with grade 2 disability (G2D) in Bangladesh continue to experience diminished well-being despite effective multidrug therapy. Community-based rehabilitation (CBR) integrated with primary health care could offer accessible, comprehensive services addressing their needs, but its acceptability and implementation challenges in Bangladesh remain poorly documented. This mixed-methods study, conducted February-July 2023, examined CBR acceptability and delivery barriers among leprosy patients with G2D. The quantitative component used a cross-sectional design, conveniently sampling 356 leprosy patients with G2D from government and private hospitals across three divisions; face-to-face interviews captured sociodemographic characteristics, disability status, CBR acceptability, and service challenges. The qualitative component comprised 15 key informant interviews with leprosy service personnel and five focus group discussions involving 8–10 G2D patients each, analyzed using deductive thematic analysis. Participants were predominantly male (65.2%) (mean age: 48.0 ± 13.7 years) with majority having foot disability (50.8%). CBR acceptability was remarkably high, with universal agreement on rehabilitation services at health complexes and community clinics, assistive technology, counseling facilities, and self-help groups. Nearly all agreed counseling would improve quality of life (99.4% [95%CI: 97.9-99.9]) and community awareness would reduce emotional distress (99.7% [98.4-99.9]), though only 67.4% [62.3-72.3] could independently perform ulcer self-care despite disability management training. Major challenges included healthcare workers’ reluctance (78.4% [73.7-82.5]), inadequate equipment (59.5% [54.3-64.7]), and insufficient training (5.6% [3.5-8.5]). Qualitative findings corroborated these results, highlighting infrastructural deficiencies, discontinuous training, competing health priorities, long travel distances, poor coordination with social welfare authorities, resource constraints, and misconceptions about transmission. Both patients and providers strongly supported CBR integration into primary health care, citing grassroots accessibility, peer learning, and stigma reduction, and recommended multi-level strategies engaging local government, religious institutions, media, and schools. Strong community acceptability supports CBR integration into primary health care, though achieving zero disability goals requires stigma reduction, healthcare worker capacity building, resource allocation, and intersectoral coordination.

## Introduction

For hundreds of years, leprosy patients have been a victim of social stigma leading to exclusion from social and economic life. The tantalizing and insensitive reactions from family members, peers, and community people affect every facet of their lives. Despite the elimination of leprosy as a public health problem, leprosy cases continue to occur and, in many cases, cripple the victim. Around 200,000 new cases of leprosy are reported every year [1]. In 2024, an estimated 172,717 incident cases of leprosy were reported worldwide [2]. Of them, 9,153 cases (5.3%) had grade 2 disability (G2D) [3]. But the development of disability could have been prevented, or its progression could have been halted through rehabilitation programs at the community level [4].

Community-based rehabilitation (CBR) was first initiated by the World Health Organization (WHO) following the International Conference on Primary Health Care in 1978 and the resulting Declaration of Alma-Ata [5]. People with disabilities, their families, and communities can work together as part of the CBR strategy to establish their rights and get access to the necessary health, vocational, economic, and social services. The Dhaka Community-Based Rehabilitation Project (DCBR) demonstrated that the community-driven development strategies can play an appropriate and gap-filling role in rehabilitating the leprosy-affected people in the working areas where there is a relatively high prevalence of leprosy [6].

The Global Leprosy (Hansen’s disease) Strategy 2021–2030 aims to achieve a target of zero disease, zero disability, and zero discrimination by 2030 [7]. To achieve these targets, the CBR needs to be integrated with Primary Health Care (PHC) in Bangladesh. Thereby, rehabilitation services can be easily accessed by the disabled leprosy patients. As CBR consists of medical, social, psychological, and vocational rehabilitation, it can provide comprehensive care to leprosy patients with G2D.

However, very few studies explored the adaptability of CBR in Bangladesh. This study aimed to explore the acceptability and challenges of Community-Based Rehabilitation (CBR) at the primary care setting for leprosy patients with G2D in Bangladesh.

## Methods

### Study design, place, and population

We applied a mixed method (quantitative and qualitative) approach to conduct this study between February and July 2023. In the quantitative part, a cross-sectional design was adopted where diagnosed cases of leprosy patients with G2D at public and private specialized leprosy hospitals were approached. Patients of any age with diagnosed type II disability, irrespective of treatment status, were considered for inclusion. Patients with cognitive impairment, speaking difficulty, or unwillingness to participate in the study were excluded. In the quantitative part, we aimed to describe the acceptability and challenges associated with CBR, and the study design did not involve any exploration of associated factors.

The sample size was calculated using the formula,


$$\begin{array}{c} \text{n}=\frac{Z^{\text{2}}P(\text{1}-P)}{d^{\text{2}}} \end{array}$$

Due to the lack of a study exploring the acceptability of CBR among leprosy patients with disability, taking an assumed 50% prevalence (p), a standard normal deviate at 5% confidence interval of 1.96 (Z), and a margin of error (d) of 5%, the calculated sample size (n) was 384. However, a total of 356 patients could be conveniently selected within the stipulated time, where 181 were from the Dhaka division (level two administrative zone), 157 from the Rangpur division, and 18 were from the Mymensingh division. The locations were chosen because of the high burden of leprosy [8], the presence of specialized leprosy centers, and the convenience of data collection. Patients coming to the hospital for admission or follow-up outdoor visits were consecutively selected. Registered patients who did not come to the hospital at their regular follow-up visit were reached over the phone and requested to come to the hospital. They were approached for inclusion upon arrival. None of the patients approached refused participation.

For the qualitative part, 15 key personnel related to leprosy services (including supervisor, program manager, and helping staff) were selected for Key Informant Interviews (KIIs) (S1 Table). In addition, five focus group discussion sessions (FGDs) were conducted with 8–10 participants each. Four FGDs included G2D Leprosy patients and, and one FGD involved nurses with experiences in leprosy management. Participants were selected using purposive sampling to ensure inclusion of individuals with relevant knowledge and experience related to the study topic. A maximum variation approach was applied to capture diverse perspectives across different roles in sociodemographic and service contexts. Selection criteria for key informants included professional role, years of experience, and involvement in service delivery, while FGD participants were selected based on eligibility criteria such as recent experience with the service, residence in the study area, and willingness to participate.

### Data collection technique

#### Quantitative part.

For the quantitative part, data were collected using a semi-structured questionnaire from leprosy patients through face-to-face interviews. The questionnaire was developed through a literature review [9–13] and expert consultations. It was pretested among five leprosy patients with grade II disability in a rural community in Dhaka, who were not included in the main dataset. The questionnaire asked patients about sociodemographic information, family history of leprosy, disability status, ongoing treatment modality, acceptability of CBR among patients, and challenges felt by patients while using existing services.

**Acceptability of CBR:** The acceptability questions were divided into two sections. These explored the acceptability of CBR based on primary health centers, and of CBR based on the community neighborhood (i.e., village level). The former consisted of 11 questions with “yes” and “no” answers, and the latter had 15 questions with “yes” and “no” answers.

**Challenges of the existing service:** The challenge-related question focused on patients’ experience of using existing support services with respect to rehabilitation. The questions were framed to determine the feasibility of building a CBR center at the healthcare facility or in the community using existing facilities. A total of 6 questions were asked with “yes” and “no” answers.

#### Qualitative part.

For the qualitative part, KII and FGD guidelines were used. The guidelines mainly focused on issues related to the rehabilitation of leprosy patients with G2D. The KII guideline explored the current situation of rehabilitation services for G2D leprosy patients with respect to treatment, infrastructure, and training. The FGD guideline focused on exploring the views of G2D leprosy patients on the possibilities of CBR self-care, self-help group, and ulcer care in rehabilitation, and challenges associated with the utilization of existing community resources. Data was collected by two trained public health graduates (one female and one male) from medical and dental backgrounds. They did not have any prior relationship or interactions with the interview participants, reducing potential bias. The interviewers used probes to add clarification and to keep the discussion on topic. The KII interviews were conducted through prior appointments and at the informants’ room in their institution. The purpose of the study was explained, and permission was taken for audio recordings before the interview. Each interview took around 30–40 minutes to complete. Each FGD was conducted by two public health graduates simultaneously. One as the facilitator and another as the note taker. FGDs with female patients and nurses were facilitated by the female interviewer. The interview took place in a calm and quiet room in the respective hospital. Permission was taken for audio recording. Discussion lasted between 60 and 70 minutes. Interviews were conducted in Bangla using translated KII and FGD guidelines. The audio records were transcribed verbatim by the interviewers. The transcription was then translated into English by the interviewers and verified by an expert English speaker. Interviewers used interview guidelines to ensure objectivity and uniformity during the procedure.

### Data analysis

#### Quantitative analysis.

For the quantitative part, descriptive statistics were presented using frequency (percentage) or mean (standard deviation) where appropriate. For key proportions a Clopper-Pearson (exact) intervals (95% Confidence Intervals) were reported. No imputation was required, as there were no missing data. All the statistical analysis was performed using the statistical software SPSS version 26.

#### Qualitative analysis.

For the qualitative part, a deductive thematic analysis approach was used, guided by a pre-specified analytical framework. Themes and subthemes were developed a priori based on the study objectives, conceptual framework, and literature review [4,7,14–20]. Data from KIIs and FGDs were systematically coded and organized according to this framework to explore participants’ perspectives on the predetermined domains of interest. Data was coded manually by two investigators (KA and AM), and any discrepancies were resolved through consultation with the third investigator (FK). To ensure trustworthiness, regular team debriefings were conducted throughout data collection and analysis to reflect on thematic conformity and minimize individual bias. Triangulation was achieved by comparing findings across different participant groups and data sources (interviews and FGDs). Field notes were maintained to support the interpretation of the data.

#### Integration of qualitative and quantitative findings.

This was undertaken at the interpretation stage using a triangulation approach. Quantitative results provided the overall patterns and magnitude of key outcomes, while qualitative data from interviews and FGDs were used to explain and contextualize these findings. Areas of convergence (agreement), complementarity (where qualitative insights added depth), and divergence (where findings differed) were systematically examined.

### Ethical considerations

The ethical clearance for the study was obtained from the Institutional Review Board of the National Institute of Preventive and Social Medicine (NIPSOM) (Memo no-NIPSOM/IRB/2023/01). Informed written consents were taken from all participants before inclusion. All the procedures were conducted following the guidelines of the Declaration of Helsinki.

## Results

### Quantitative findings

#### Participant characteristics.

The study included 356 participants with a mean age of 48.0 years (SD = 13.7). The majority were male (65.2%), married (81.7%), and illiterate (46.9%). Regarding occupation, 29.8% were unemployed, 28.7% were housewives, 23.0% were employed, and 18.5% were engaged in business. In terms of education, 32.6% had primary education, 18.5% had education up to secondary school certificate (SSC), and only 2.0% had higher secondary certificate (HSC) or above. Concerning disability status, half of the participants (50.8%) had foot disability, 28.4% had both hand and foot disability, 19.1% had hand disability only, and 1.7% had eye involvement. At the time of the study, 44.7% were receiving oral medicine, 39.3% were receiving ulcer care, and 16.0% were receiving both treatments. Treatment was primarily sought from government organizations (53.4%), followed by non-government organizations (37.6%), with 9.0% accessing both. Most participants reported good experiences with rehabilitation (59.6%), while 36.8% reported average experiences and 3.7% reported bad experiences (Table 1).

**Table 1:** Sociodemographic profile, disability status, and treatment-related characteristics of the participants.

Attribute	n (%) or mean (SD)
**Age (years)**	48.0 (13.7)
**Sex**	
Female	124 (34.8)
**Marital status**	
Married	291 (81.7)
Unmarried	35 (9.8)
Widower	23 (6.5)
Divorce	6 (1.7)
Separate	1 (0.3)
**Occupation**	
Housewife	102 (28.7)
Job	82 (23.0)
Business	66 (18.5)
Unemployed	106 (29.8)
**Education level**	
Illiterate	167 (46.9)
Primary	116 (32.6)
Up to SSC	66 (18.5)
HSC and above	7 (2.0)
**Disability status**	
Hand disability	68 (19.1)
Foot disability	181 (50.8)
Both hand and foot disability	101 (28.4)
Eye involvement	6 (1.7)
**Ongoing treatment**	
Oral medicine	159 (44.7)
Ulcer care	140 (39.3)
Both	57 (16.0)
**Place of treatment**	
Government organizations	190 (53.4)
Nongovernment organizations	134 (37.6)
Both	32 (9.0)
**Experience with rehabilitation**	
Good	212 (59.6)
Average	131 (36.8)
Bad	13 (3.7)

#### Acceptability of community-based rehabilitation.

Acceptability of community-based rehabilitation was remarkably high (Table 2). All participants (100%) agreed that rehabilitation services should be available in health complexes and community clinics near home, and that such services would reduce stigmatization. Universal agreement was observed regarding the benefits of counseling facilities, assistive technology, and the involvement of field health workers in awareness and detection activities. Nearly all participants agreed that counseling would improve quality of life (99.4% [95%CI: 97.9 – 99.9]) and that community awareness would reduce emotional distress (99.7% [98.4 – 99.9]). All participants recognized the value of self-help groups in helping disabled persons and their families, improving communication and mental development, providing income opportunities, and enhancing accountability when located in health complexes. While all participants reported being taught disability management and agreed on the need for regular ulcer care, lower proportions reported being able to take care of their ulcers (67.4% [62.3 – 72.3]) and agreed that self-care training should be provided in various settings (71.9% [66.9 – 76.5]).

**Table 2:** Acceptability of community-based rehabilitation (n = 356).

Characteristic	n	% (95%CI)
***Acceptability of CBR based on PHC***		
It is better to have rehabilitation services in health complexes near home.	356	100 (99 – 100)
Community clinics should have primary rehabilitation services.	356	100 (99 – 100)
If there are leprosy rehabilitation services at a health complex or community clinic, people around you will know and you will be less stigmatized.	356	100 (99 – 100)
Family members or neighbors will not push you away if counseling facilities are provided at the health center.	356	100 (99 – 100)
Medical leprosy rehabilitation is easier for you if you stay at a health complex.	356	100 (99 – 100)
Using assistive technology will make your life easier.	356	100 (99 – 100)
Using assistive technology will improve your quality of life.	356	100 (99 – 100)
Your quality of life would be better if you had counseling at a health complex or community clinic.	354	99.4 (97.9 – 99.9)
Community awareness would have reduced your emotional distress.	355	99.7 (98.4 – 99.9)
It would be good to create community awareness in health centers.	356	100 (99 – 100)
It is better to engage in awareness, detection, supervision, and reporting towards field health workers.	356	100 (99 – 100)
***Acceptability of CBR centers in the neighborhood***		
Self-help groups help disabled people.	356	100 (99 – 100)
Self-help groups also help the family members	356	100 (99 – 100)
Self-help groups make life easier	356	100 (99 – 100)
Self-help groups increase communication with each other, which helps mental development.	356	100 (99 – 100)
It would provide opportunities to make some money	356	100 (99 – 100)
Disabled persons can be empowered through self-help groups that are supervised by the Ministry of Social Welfare.	356	100 (99 – 100)
One would be financially sound through rehabilitation	356	100 (99 – 100)
Accountability of self-help groups or records of their activities would be better if they were in health complexes.	356	100 (99 – 100)
The participant was taught to manage their disability.	356	100 (99 – 100)
It would be good to have a training in the health complex/community clinic/in the home/neighborhood to teach the disabled self-care.	256	71.9 (66.9 – 76.5)
The participant can take care of an ulcer.	240	67.4 (62.3 – 72.3)
It would have been better to have regular early ulcer care at the health complex or community clinic.	356	100 (99 – 100)
The health center will actually increase mental alertness.	356	100 (99 – 100)
Depression will go away through self-help group visits	356	100 (99 – 100)
A self-help group visit would help gain confidence	356	100 (99 – 100)

#### Challenges with existing support services.

The most frequently reported challenge was healthcare workers’ reluctance to rehabilitate leprosy patients (78.4% [73.7 – 82.5]), followed by inadequate equipment in health centers (59.6% [54.3 – 64.7]). Other challenges included a lack of manpower and funding for assistive devices (35.4% [30.4 – 40.6]), insufficient funds (24.16% [19.8 – 28.9]), inadequate manpower in health complexes (12.6% [9.4 – 16.6]), and lack of healthcare worker training (5.6% [3.5 – 8.5]) (Table 3).

**Table 3:** Challenges associated with existing support services.

Characteristic	n	% (95%CI)
Healthcare workers are very reluctant to rehabilitate leprosy patients	279	78.4 (73.7 – 82.5)
The health center is not well-equipped for leprosy rehabilitation	212	59.6 (54.3 – 64.7)
Lack of training of the healthcare workers	20	5.62 (3.5 – 8.5)
Lack of manpower in health complexes	45	12.6 (9.4 – 16.6)
Lack of funds	86	24.2 (19.8 – 28.9)
Lack of manpower and funding to assist with devices	126	35.4 (30.4 – 40.6)

### Qualitative findings

**Fig 1:**
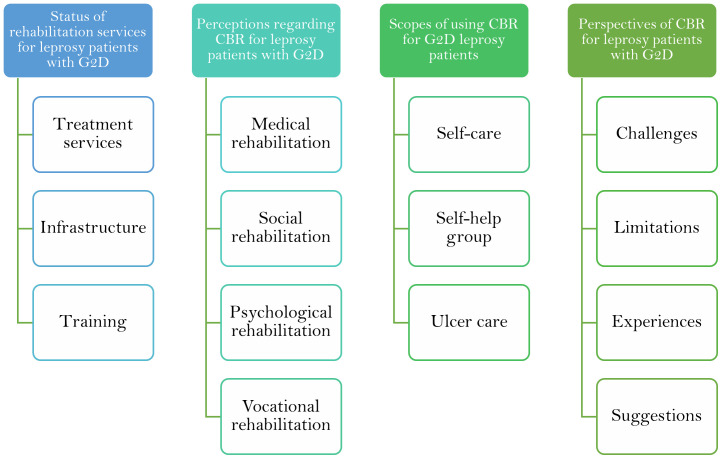
Thematic framework used in the qualitative study.

The qualitative analysis of key informant interviews (KIIs) and focus group discussions (FGDs) revealed several sub-themes within the four themes explored in this study (Fig 1). Overall, the qualitative findings revealed integrated GO-NGO leprosy services with adequate infrastructure but limited rehabilitation training. Participants valued community-based rehabilitation across medical, social, psychological, and vocational domains, supporting self-help groups and ulcer care. Key challenges included stigma, competing TB priorities, poor coordination, and access barriers, with strong calls for localized, awareness-driven services.

#### Theme 1: Status of rehabilitation services for leprosy patients with grade 2 disability.

This theme encompassed three subthemes: treatment services, infrastructure, and training.

**Treatment services:** Participants described the integration of government and non-government organizations in leprosy service delivery. One key informant noted:

*“From 2017, GO-NGOs have been integrated. NGOs were merged with GOs and are now working together. NGO field staff and GO field staff, like TLCA [Tuberculosis and Leprosy Control Assistant], and LCA [Leprosy Control Assistant] work together.” [KII-04]*

Another informant emphasized the ongoing struggle in treatment delivery: *“We are struggling to cope with the rehabilitation service delivery process for grade 2 leprosy patients” [KII-01]*.

The presence of referral systems was mentioned by key informants. Patients were usually referred to specialized centers, including Dhaka Medical College Hospital and Shaheed Suhrawardy Medical College Hospital. Funding support is also available for those who cannot afford medicines. One informant noted that “*all medical centers have social welfare centers*” and that “*social welfare centers provide [fund] the medicine if needed*” for those who cannot afford ulcer care medications [KII-04].

**Infrastructure:** While infrastructure was generally described as adequate, with one informant stating that “*Health infrastructure is good. All NGOs use government infrastructure and manpower*” [KII-02], gaps in rehabilitation facilities were apparent. A key informant explained: *“In the health complex, rehabilitation facilities are not available. Where TLCA is absent, HA [Health assistant] or AHI [Assistant Health Inspector] provides the service. Health complex could provide ulcer care...”* [KII-02].

**Training:** Training gaps were identified as a significant challenge. Participants reported: *“Training on leprosy is given, but not continuously. No training was given on rehabilitation”* [KII-11] and *“There is little training on counseling in psychological rehabilitation”* [KII-07].

#### Theme 2: Perceptions regarding community-based rehabilitation for leprosy patients with G2D.

This theme explored the views of Key Informants and G2D leprosy patients regarding CBR services in primary health care settings. They examined the rehabilitation in four domains: medical, social, psychological and vocational.

**Medical rehabilitation:** Participants emphasized the potential benefits of providing services at the grassroots level. One key informant linked the placement of rehabilitation services at the community level with the opportunity to initiate medical rehabilitation:

*“Since community clinic services are at the grassroots level, they have it within their accessibility. If the service is a little geared up, then we can provide medical rehabilitation from the beginning”* [KII-12]

**Social rehabilitation:** Leprosy patients recognized the benefit of counselling in removing irrational fear from society. FGD participants suggested that *“Upazila health complex or community clinic can provide first aid” and that “counseling can remove the fear that people have about this disease, that this disease is not contagious”* [FGD-01, FGD-03]. One key informant emphasized the importance of community awareness in the social rehabilitation of patients, saying, *“There is no alternative to community awareness”* [KII-02]. Another key informant stated, *“Through social counseling, it can be taught that leprosy is not a contagious disease. It gets better with treatment.”* [KII-07]

FGD participants also highlighted the importance of family counseling in removing social stigma:

*“Family counselling is important. If the patient is deprived of family, then the surrounding neighbors also ignore them.”* [FGD-02]

**Psychological rehabilitation:** Psychological rehabilitation was possibly the least understood type of rehabilitation among the key informants and patients. None were able to elicit the importance of psychological rehabilitation for these chronic sufferers. However, one informant identified the need for a dedicated and trained counsellor in the primary health care centers for this purpose: *“If psychological rehabilitation is to be established in the Upazila Health Complex (UHC), one counsellor is needed”* [KII-08].

**Vocational rehabilitation:** The majority of the key informants agreed on vocational rehabilitation for leprosy patients with G2D. One participant recognized that not all work would be appropriate for G2D leprosy patients. However, training can be given regarding the specific type of work the patients are able to do: *“Those work the patient can do, we can give training on those...”* [KII-05].

One key informant suggested providing disability allowance to meet the financial needs of these patients. However, the importance of verification to ensure proper distribution of the allowance was also emphasized. He said,

*“.. In Upazila, through UHFPO [Upazila Health and Family Planning Officer] authorization, a disability allowance is recommended. So, for grade 2 disability, a certificate can be given by UHFPO…”*[KII-07]

Patients showed interest in vocational work, as they are unable to find regular jobs and didn’t want to beg for money from other people. One patient explained: *“We can not do any work now a days, since we are affected by the disease. Suitable vocational work would have been really great for us. We could have earned some money ourselves rather than begging for money.”* [FGD-06]

#### Theme 3: Scopes of using CBR services for leprosy patients with G2D.

This theme explored the feasibility and potential impact of CBR services, including self-care, self-help groups, and ulcer care in rehabilitation.

**Self-care:** Participants described existing self-care education efforts. One key informant explained: “Field staff visit grade 2 disabled leprosy patients’ house and teach them self-care” [KII-02].

FGD participants suggested that *“if the community clinics created groups and the group could help new patients learn basic self-health care”* [FGD-04, FGD-05]. Training in health facilities was also mentioned: *“in UHC [Upazila Health Complex, training can be given on self-care, [like] sessions are given to prevent burns”* [KII-12].

**Self-help groups:** Both key informants and patients emphasized the importance of creating self-help groups in the community for G2D leprosy patients. One key informant described how the formation of self-help groups helps patients learn self-care.

*“We have started to give community-based rehabilitation (CBR) in 8 upazila. We make the family aware. We organize leprosy patients into a group and teach them how to take care of ulcers. We give demos on how to take care of ulcers. But mostly the demo is given by an old leprosy patient who is experienced with ulcer care or disability.”* [KII-02]

FGD participants expressed strong support for self-help groups based on the community [FGD-02, FGD-04, FGD-05].

**Ulcer care:** Regarding ulcer care, the key informants described how they provide ulcer and stated that ulcer care should be provided at the upazila health complex and at the house level. However, they stressed the need for customized footwear and the need to train the service providers working in the field.

“*Field staff visit their [patient’s] house and teach ulcer care. [But] They need training.”* [KII-02]

#### Theme 4: Perspectives of CBR for leprosy patients with G2D.

This theme explored the challenges, limitations, experiences, and expectations related to CBR among key informants and patients.

**Challenges:** Competing priorities, distance, lack of coordination, and the prevailing stigma were identified as key challenges by the participants. The high burden of tuberculosis is given priority in terms of service delivery, leading to the care for leprosy being neglected. One key informant highlighted the competing priorities in resource allocation:

*“As the death rate is low in leprosy patients, it is now a less important concern. In TB, death rate is high (4-5%). For that reason, TB is now the primary concern.”* [KII-02]

Patients who are coming to the existing care facilities from traversing a long distance felt the need for community-based rehabilitation facilities the most. FGD participants expressed frustration with travel distances:

*“We have to come so far each time, people keep asking where we are going and everything. If these health organizations could provide us with treatments, we would not have to come this far away from home seeking treatment. And we could have easily taken our treatment at a much lower cost. If we could take our treatment from health complexes and community clinics within our locality, that would have been much easier for us.”* [FGD-04, FGD-05]

Coordination problems with social welfare authorities and provider reluctance were noted, with FGD participants stating: *“Lack of coordination with social welfare authority. People don’t take it easy. Providers should be trained in such a way that they do not hesitate to provide services, and do not show reluctance”* [FGD-01, FGD-03].

Stigma related to leprosy-associated disability is still prevailing in society. Multiple participants stressed the need to reduce stigma related to leprosy. One key informant clarified:

*“It is not like a highly infectious disease, COVID-19. Leprosy does not spread unless a patient is in contact with another person for over 20 hours. So, leprosy patients need not to be stigmatized by the general public, or even by [some] health care providers, because leprosy is completely curable with medicine.”* [KII-07]

An FGD participant added: *“It would be good if everyone in the society were made aware that this disease is not contagious”* [FGD-03].

**Limitations:** Lack of funds, human resources, and access barriers due to physical disability were identified as the main limitations in relation to CBR. Administrative challenges were identified underscoring the lack of dedicated manpower for the CBR: *“Challenges are faced in planning and policy making of leprosy control. [For example,] The absence of leprosy-dedicated doctors. In the administrative level, there is a vacancy of posts”* [KII-02].

Patients have difficulty accessing services because of their physical disability and distance. One Key Informant stated: *“There is disability of the legs and the problem of walking, …they do not always want to come to the health facility”* [KII-04].

**Experience:** Key informants shared their experience working in the healthcare of G2D leprosy patients. They felt happy seeing patients improve and had deep empathy for their suffering. But they also noted noncompliance from patients. As one community development facilitator described: “*Grade 2 disabled patients are very helpless. They do not take medicine and care for ulcers properly. Even with the difficulties, they continue the work which is harmful for them.”* [KII-05]

**Suggestions:** Participants offered several suggestions for strengthening rehabilitation services. One key informant proposed:

*“[We] have to build community awareness, involve field workers, including the local government, so that the rehabilitation service can be ensured. Media can play a vital role. Through school health, flash card with TB leprosy message could be distributed. In 5 year, plans of HPNSP [Health Population Nutrition Sector Program], more focus could be given on leprosy.”* [KII-07]

Strengthening coordination mechanisms was also suggested, with mention of the Leprosy TB Coordinating Council (LTCC) as a model for more vigorous programming under the National Leprosy Program. FGD participants emphasized the need for action and early intervention:

*“We need actions rather than planning, because we have seen much planning, but actions are scarce.”* [FGD-04, FGD-05]

Patients suggested using health complexes, social groups, and mosques to increase awareness and dispel misconceptions about leprosy to prevent its progression and the development of crippling disability.

*“If people were aware of these diseases, then this disease could have been arrested at a very early stage. And our disabilities would have been much less. If health complexes and social institutions like mosques were used to build awareness and reduce false stigma, it would have been good. Due to a lack of proper awareness, sometimes the disease is unnoticed even for many years before crippling us.”* [FGD-04, FGD-05]

The desire for localized services was reiterated: *“It would be great if the same service we get here can be provided closer to home”* [FGD-01].

## Discussion

This mixed-methods study explored the acceptability and challenges of community-based rehabilitation for leprosy patients with grade 2 disability in Bangladesh. The triangulation of quantitative and qualitative findings reveals strong community acceptance for CBR integration into primary health care, alongside significant systemic barriers that must be addressed to achieve the WHO’s Global Leprosy Strategy goal of zero disability by 2030 (WHO, 2021).

### High acceptability and strong demand for community-based rehabilitation

The quantitative findings demonstrated remarkably high acceptability of CBR, with universal agreement (100%) among participants regarding the need for rehabilitation services in health complexes and community clinics. This finding was strongly corroborated by qualitative data, where both key informants and FGD participants emphasized the accessibility benefits of grassroots-level services. This underscores a genuine community readiness for CBR integration.

The near-universal acceptability observed in this study aligns with recent evidence demonstrating that integrated approaches to leprosy care within primary health care systems effectively reduce stigma and improve service utilization [15,21]. Willis and colleagues’ systematic review identified integration of leprosy within the health system as one of six effective interventions for reducing leprosy-related stigma. Our qualitative findings support this mechanism, as participants expressed that having services at community clinics would reduce the social exposure associated with traveling long distances to specialized leprosy facilities.

### The role of stigma reduction through integration

Both quantitative and qualitative data revealed stigma as a central concern influencing CBR acceptability. The importance of stigma reduction is well-documented in recent literature. Willis et al. (2024) found that multiple intervention categories, including information, education, and communication (IEC), community-led projects, and socioeconomic rehabilitation, successfully reduce leprosy-related stigma. However, their review noted that evidence remains restricted to three Asian countries (Nepal, India, and Indonesia), highlighting the importance of studies like ours that examine the opportunities of community based rehabilitation initiatives in Bangladesh. Recent research emphasizes that stigma not only affects access to diagnosis and treatment but also violates civil, political, and social rights of affected persons [22].

Our findings suggest that integration of CBR into primary health care could address what Tegene et al. [23] describe as the ongoing challenge of delayed diagnosis and persistent stigmatization despite effective treatment availability. The qualitative data from our study revealed that participants believed counseling at community facilities could “remove the fear that people have about this disease, that this disease is not contagious,” directly addressing the misconceptions that perpetuate stigma.

### Self-help groups and socioeconomic rehabilitation

Leprosy patients showed a universal recognition (100%) of self-help groups’ value in helping disabled persons, improving mental development, and providing income opportunities. Qualitative findings enriched this understanding by revealing the mechanisms through which self-help groups operate. This peer-to-peer learning model aligns with recent evidence on self-help interventions for leprosy. In the Dhaka Community-Based Rehabilitation Project by the Leprosy Mission International [6] self-help groups have been proven to reduce the experience of stigma, increase well-being, and enhance household economic performance. Community-based vocational training programs in Nepal have demonstrated success through small-scale business grants and scholarships for leprosy-affected individuals. In Nepal, a self-help intervention called IMPACT (Integrated Mobilization of People for Active Community Transformation) showed that participants experienced improvements in health outcomes, social participation, and economic status through structured self-care and self-help activities [24].

However, our study revealed a critical gap between knowledge and practice. While all participants (100%) reported being taught disability management, only 67.42% could took care of their ulcers. This discrepancy suggests that knowledge transfer alone is insufficient. As one key informant noted that leprosy patients with G2D tend not to comply with medicine. Hence, a comprehensive support system is essential that addresses not only knowledge but also the socioeconomic factors that prevent proper self-care.

### Critical gaps in rehabilitation services

We found a considerable delivery service gap. Quantitatively, 78.37% of participants reported healthcare workers’ reluctance to rehabilitate leprosy patients, and 59.55% cited inadequate equipment. The key informants pointed out the absence of manpower and funds in service, highlighting the struggle in service delivery. These findings resonate with recent evidence from Ethiopia, where Tegene et al. [23] found that even in specialized leprosy centers, rehabilitation services suffer from a lack of trained staff and necessary equipment. Similarly, research in Ethiopia by Abdela et al. [14] revealed that rehabilitation centers, though established, remain non-functional due to insufficient professional support and equipment. This parallel between our findings and those from other endemic countries suggests systemic issues in resource allocation for leprosy rehabilitation across low- and middle-income settings.

The training gap identified in our study is particularly concerning. This training deficit likely contributes to the healthcare worker’s reluctance documented quantitatively. Recent literature emphasizes that when rehabilitation becomes part of community development, workers need different competencies than those required in vertical disability programs, including the ability to function as ‘change agents’ rather than merely service providers [25].

### Comprehensive rehabilitation: Medical, psychological, social, and vocational dimensions

Our qualitative analysis revealed varying levels of understanding and implementation across the four rehabilitation domains. While medical and social rehabilitation received substantial attention from participants, the need for psychological rehabilitation was not well understood. This finding is significant in light of recent research emphasizing the mental health impacts of leprosy. Recent studies have documented that patients frequently experience depression, anxiety, and social isolation, which not only diminishes quality of life but also impedes recovery [26]. The WHO Global Leprosy Strategy 2021–2030 [7] explicitly includes combating stigma and ensuring mental wellbeing as one of its four strategic pillars, yet our findings suggest that psychological rehabilitation remains the least emphasized component in Bangladesh’s leprosy services.

Regarding vocational rehabilitation, the triangulation of data revealed both need and opportunity. All participants agreed that rehabilitation would lead to financial soundness. In the focus group discussions, patients expressed a clear desire for economic empowerment: Recent evidence from India demonstrates that vocational training centers providing skills in various trades (computing, mechanics, farming, sewing, etc.) with industry linkages achieve successful employment outcomes for persons affected by leprosy [27]. However, as key informants in our study noted, such vocational programs would require coordination with the Ministry of Social Welfare and careful assessment of patients’ functional capabilities.

### Resource constraints and competing priorities

A critical finding from our triangulated analysis concerns the deprioritization of leprosy relative to other diseases. It represents a significant barrier to achieving zero disability goals. As noted in recent literature, many governments scaled down leprosy programs following the elimination of leprosy as a public health problem, mistakenly believing the disease no longer required attention [28]. However, even where active national programs exist, they typically focus solely on case-finding and treatment, with minimal attention to disability and stigma. The WHO’s 2024 epidemiological report [29] shows that approximately 182,815 new cases were detected globally in 2023, with Bangladesh among the countries reporting 3622 new cases, indicating continued transmission that will generate future disability if not addressed comprehensively.

### Integration strategies and implementation pathways

Coordination among local government, religious institutions, social organizations and media was an important suggestion that came out of the interviews for a successful implementation of community-based rehabilitation of leprosy patients with G2D. Participant recommended that school-based and mosque-based approaches to increase awareness regarding leprosy. Fastenau et al. [30] argue that integrating community engagement in zero leprosy efforts creates a pathway to sustainable early detection, control, and elimination. A Comprehensive Rural Health Project in India demonstrated that integrated approaches to leprosy care within primary health services resulted in virtually non-existent social stigma in communities, compared to high levels of stigma where vertical approaches were used [15]. This suggests that Bangladesh could benefit from similar integration strategies, leveraging existing health infrastructure while ensuring adequate training and resources.

### Study limitations and strengths

Several limitations of the study warrant consideration. The convenience sampling approach may have introduced selection bias and limited generalizability to all leprosy patients with type 2 disability. The calculated sample size could not be achieved, reducing the precision of the estimates by a trivial 0.18%. The very high levels of reported acceptability, including instances of universal agreement, should be interpreted cautiously. These findings may reflect ceiling effects, potential measurement limitations, and the framing of questions toward positive aspects of the intervention. As the interviews were conducted at the health facilities by public health graduates and because of the sensitive nature of the topic, the possibility of acquiescence and social desirability bias could not be excluded. As a result, the instrument may not have fully captured more nuanced views. The cross-sectional design precludes causal inferences about the relationship between CBR implementation and outcomes. Future longitudinal studies with larger, probability-based samples would strengthen evidence for policy decisions. The deductive thematic analysis limits the discovery of novel themes from the qualitative interviews. A phenomenological approach could be taken in further qualitative studies.

Despite these limitations, the combination of quantitative and qualitative approach strengthens confidence in our findings. The convergence between high quantitative acceptability rates and rich qualitative narratives about access, stigma, and service needs provides robust evidence for policy action. The study contributes to the limited literature on CBR for leprosy in Bangladesh and provides practical insights for implementing the National Leprosy Program’s goals aligned with WHO’s global strategy.

## Conclusion

This study demonstrates strong community acceptability for integrating CBR into primary health care in Bangladesh, with universal recognition of benefits across medical, social, and economic dimensions. However, significant implementation barriers exist, including healthcare worker reluctance, inadequate infrastructure, training deficits, and resource constraints driven by competing health priorities. The triangulation of quantitative and qualitative findings reveals that successful CBR implementation requires a comprehensive approach addressing not only service availability but also stigma reduction, community awareness, healthcare worker capacity building, and intersectoral coordination with social welfare systems.

## Supporting information

S1 Table Supplementary tables. (DOCX)

S1 Data Dataset. (SAV)

S1 STROBE Checklist The STrengthening the Reporting of OBservational studies in Epidemiology (STROBE) checklist for cross-sectional study. (DOCX)

S2 SRQR Checklist The Standards for Reporting Qualitative Research (SRQR) checklist for qualitative study. (DOCX)
