# Supplementary material for: Acceptability and challenges of community-based rehabilitation (CBR) of leprosy patients with grade-2 disability in Bangladesh
Source: PLOS Glob Public Health. 2026 Aug 7;6(8):e0006994. doi: 10.1371/journal.pgph.0006994 (PMC13450763; doi:10.1371/journal.pgph.0006994)
Supplement: Supplementary file 2 [file pgph.0006994.s001.docx]

**S1 Table. List of key informants interviewed in the study (n=15)**

| **Sl** | **Workplace** | **Direct management** | **Helping**  **staff** | **Supervisor** |
| --- | --- | --- | --- | --- |
| **01** | Line director (TBL-ASP) | 01 |  |  |
| **02** | Program manager | 01 |  |  |
| **03** | Deputy Program manager | 01 |  |  |
| **04** | Assistant Director, Leprosy Hospital, Dhaka | 01 |  |  |
| **05** | RMO, Leprosy Hospital, Dhaka | 01 |  |  |
| **06** | Program coordinator DBLM hospital | 01 |  |  |
| **07** | Nursing supervisor, DBLM hospital |  |  | 01 |
| **08** | Danish Bangladesh | 01 |  |  |
| **09** | Community Field worker |  | 02 |  |
| **10** | TBLM |  | 01 |  |
| **11** | UHFPO- Nilphamari sadar |  |  | 01 |
| **12** | UHFPO- Kamrangichar |  |  | 01 |
| **13** | UHFPO- Kaliakair |  |  | 01 |
| **14** | Leprosy Mission Bangladesh |  |  | 01 |

DBLM: Danish Bangladesh Leprosy Mission; RMO: Resident Medical Officer; TBLM: TBL-ASP: TB- Leprosy and AIDS/STD Program; UHFPO: Upazila Health and Family Planning Officer
