# Supplementary material for: Acceptability and challenges of community-based rehabilitation (CBR) of leprosy patients with grade-2 disability in Bangladesh
Source: PLOS Glob Public Health. 2026 Aug 7;6(8):e0006994. doi: 10.1371/journal.pgph.0006994 (PMC13450763; doi:10.1371/journal.pgph.0006994)
Supplement: Supplementary file 6 [file pgph.0006994.s008.docx]

Dear Editor,

We want to thank the reviewers again for the feedback. Herein, we have included a one-by-one response to each comment that is placed immediately below the comment.

Regards,
Dr. Khursheda Akhtar

Reviewer #1: The mixed-methods design yields robust evidence of high acceptability (often 100%) alongside practical barriers, aligning well with PLOS Global Public Health's scope on global health equity

Universal agreement on acceptability items risks ceiling effects; while acknowledged, consider validating the yes/no questionnaire in future work or adding Likert scales for nuance. Sample size fell short (356/384) due to time constraints—power implications are noted, but report confidence intervals around key proportions.

**Authors’ response:** Thank you for the comment. We have reported the Clopper-Pearson (exact) intervals (95%) around the key proportions. Associated modifications were made in the result description, abstract, and methods.

In methods section Required clarifications:

Inclusion and exclusion criteria need to be defined clearly

**Authors’ response:** Thank you again. The inclusion and exclusion criteria were defined, but got buried in the middle. So we have moved it to the top of the “Study design, Place and Population” subsection and modified phrases to make it clearer. Please check lines 112 – 116.

Sample size justification (power calculation?)

**Authors’ response:** Thank you for your comment. Power calculation is valid for the hypothesis testing. As we did not do any hypothesis testing and were purely descriptive in the quantitative part. A power calculation for the sample shortfall is not applicable. However, we did estimate for our achieved samples. For example, if we could take n = 384 samples, under a standard normal deviate at 95%CI z = 1.96 and the proportion of participant who could take care of their ulcer (67.4%), the precision estimate d = 4.68%, whereas for our sample size 356, the estimate is d = 4.86. A 0.18% short fall in the precision estimate, which is a trivial loss in the precision. We have mentioned this number in the limitation section to add more specificity. Please check line 552.

Response rate

**Authors’ response:** Thank you for your comment. Hundred percent of the participants we approached for inclusion participated in the study. Hence, we did not include this in the manuscript.

In qualitative part, add information regarding data saturation for KII and FGDs.

**Authors’ response:** Thank you for your comment. As we pre-specified the number of KII and FGDs to be taken and also pre-specified the themes, we conducted all the KII and FGDs, data saturation did not need to be taken into account to shorten the number of interviews.

In results summary of qualitatative part can be presented for better readability.

**Authors’ response:** Thank you for your comment. A summary has been added at the beginning of the result. Please check lines 263 to 268.

Limitations shoud be explained futher regarding Convenience sampling bias, Hospital-based recruitment bias, Social desirability bias, Cross-sectional design limitations, Generalizability issues

**Authors’ response:** Thank you for your comment. We have mentioned all the limitations in the study limitation section with minor improvements in the revised edition.

Reviewer #2: The authors have sufficiently addressed my comments.

**Authors’ response:** Thank you very much for your time and consideration.
