# Supplementary material for: Acceptability and challenges of community-based rehabilitation (CBR) of leprosy patients with grade-2 disability in Bangladesh
Source: PLOS Glob Public Health. 2026 Aug 7;6(8):e0006994. doi: 10.1371/journal.pgph.0006994 (PMC13450763; doi:10.1371/journal.pgph.0006994)
Supplement: Supplementary file 6 [file pgph.0006994.s007.docx]

Dear editor,

We want to express our heartfelt gratitude for considering our manuscript for review. We want to thank the reviewers for their extensive and careful constructive reviews. We believe that, after the adjustment of the manuscript based on the comments, the quality of the work has improved. Herein, we have provided a point-by-point response to each comment by the reviewers. Each response is placed immediately below the comment by the reviewer.

Kind regards,
Dr. Khursheda Akhtar,

Associate professor,

Department of Public Health and Hospital Administration,

National Institute of Preventive and Social Medicine,

Mohakhali, Dhaka, Bangladesh.

**Reviewer #1:**

This is an important and timely mixed methods study assessing the acceptability and perceived challenges of community based rehabilitation (CBR) for people affected by leprosy with grade 2 disability (G2D) in Bangladesh. The work adds valuable empirical evidence from both patients and providers in a high burden setting. However, several methodological clarifications, interpretive refinements, and minor inconsistencies need to be addressed before the paper is suitable for publication.

**Authors’ response:** Thank you for the detailed review and guidance. We believe the manuscript has improved substantially because of the careful comments and suggestions.

Sampling strategy and generalizability
The quantitative component uses convenience sampling of G2D patients from selected government and NGO hospitals in three divisions. This is appropriate for a hard to reach population, but limits generalizability.

**Authors’ response:** Thank you for the comment. We have now acknowledged this in the limitation section. The relevant sentence in the limitations part at the end of the discussion section reads as follows:
“Several limitations warrant consideration. The convenience sampling approach may have introduced selection bias and limited generalizability.” (Lines 549, 550).

Please describe the inclusion and exclusion criteria explicitly (e.g., age threshold, current treatment status, cognitive/communication ability). Clarify how patients were approached and whether any refused participation

**Authors’ response:** Thank you for the comment. The inclusion and exclusion criteria was explicitly mentioned (lines 123 to 126). It now reads-

Leprosy patients of any age with type II disability, irrespective of treatment status, were considered for inclusion. Patients with cognitive impairment, speaking difficulty, and unwillingness to participate in the study were excluded.

How patients were approached and refusal was described in detail in lines 126 to 130. It now reads-

Patients coming to the hospital for admission or follow-up outdoors visits consecutively selected. Registered patients who did not come to the hospital at their regular follow-up visit were reached over phone and requested to come to hospital. They were approached for inclusion upon arrival. None of the patients approached refused participation.

Explain why these three divisions and these specific facilities were chosen (e.g., higher leprosy burden, presence of specialized services.

**Authors’ response:** Thank you for the comment. We have stated this in the methods in lines 121 -123. It now reads-

The locations were chosen because of the high burden of leprosy [5], presence of specialized leprosy centers, and the convenience of data collection.

The sample size calculation (n = Z^2 pq/d^2) assumed 50% prevalence of acceptability but the achieved sample was 356 instead of 384. Please (a) write out the full formula clearly, (b) explain the practical reasons for stopping at 356, and (c) briefly comment on the impact on precision.

**Authors’ response:** Thank you for the comment. Actually, we calculated the sample size to be 384. But, within he stipulated data collection time, we were able to reach 356 patients. We have now written the formula using equation symbols in the Microsoft Word program. The limitation of falling short of the calculated samples (i.e., the reduction in power) was written in the limitations at the end of the discussion.

Lines 114 – 118:

The sample size was calculated using the formula,

n = $\frac{Z^{2}P(1-P)}{d^{2}}$

Due to the lack of a study exploring the acceptability of CBR among leprosy patients with disability, taking an assumed 50% prevalence (p), a standard normal deviate at 5% confidence interval of 1.96 (Z), and a margin of error (d) of 5%, the calculated sample size (n) was 384.

Lines 550 – 551:

The calculated sample size could not be achieved, reducing the precision of the estimates.

Measurement of “acceptability” and “challenges”
The section describing how acceptability and challenges were measured is currently brief and partially unclear (e.g., “The acceptability was assessed for CBR based on primary health centers and on villages…”).
Please clearly specify:
The response options (e.g., Likert scale vs yes/no) for each set of items

**Authors’ response:** Thank you for the comment. The response options were now clearly described.

Lines 149 – 158:

*Acceptability of CBR*

The acceptability questions were divided into two sections. These explored the acceptability of CBR based on primary health centers, and of CBR based on the community neighborhood (i.e., village level). The former consisted of 14 questions with “yes” and “no” answers, and the latter had 16 questions with “yes” and “no” answers.

*Challenges of the existing service*

The challenge-related question focused on patients' experience of using existing support services with respect to rehabilitation. The questions were framed to determine the feasibility of building a CBR center at the healthcare facility or in the community using existing facilities. A total of 6 questions were asked with “yes” and “no” answers.

How items were developed (based on literature, expert consultation, pre testing) and whether any reliability assessment was done (even simple internal consistency).

**Authors’ response:** Thank you for the comment. The questionnaire was developed through literature review and expert consultation. It was then pre-tested among 5 patients selected from the community. We have incorporated this description in the methods with reference to some relevant literature.

Lines 143 – 145:

The questionnaire was developed through a literature review [6–10] and expert consultations. It was pretested among five leprosy patients with grade II disability in a rural community in Dhaka, who were not included in the main dataset.

Whether items were asked in a standard order and if interviewers had probes or showcards.
Given the very high levels of agreement (many items at 100%), please consider and discuss the possibility of acquiescence and social desirability bias, particularly as interviews were conducted in health facilities by health trained data collectors

**Authors’ response:** Thank you for the comment. The questionnaire used in the quantitative part did not involve any probes or show cards. Items were asked in standard order. However, the KII and FGD interview guides had probes to prevent the informants from straying from the discussion topic. We have added the following line at the end of the qualitative part of the data collection technique section:

Lines 168:

The interviewers used probes to add clarification and to keep the discussion on topic.

We have acknowledged the potential social desirability bias and the possibility of acquiescence in the limitations section. The following lines were added:

 Lines 554-556:

As the interviews were conducted at the health facilities by public health graduates, the possibility of acquiescence and social desirability bias could not be excluded.

Descriptive focus and lack of inferential analysis
The quantitative analysis is purely descriptive, which is acceptable for an exploratory study, but why were no associations were examined (e.g., acceptability by sex, age, education, type of disability).

**Authors’ response:** Thank you for the comment. As you correctly identified, because of the high acceptability of CBR-related questions among patients, inferential statistic would not be informative, and, hence, were not done. Moreover, the challenge-related questions were not formatted to reflect variation across different factors like age, sex, education, or type of disability. Rather, these reflect the overall capacity of the existing services in relation to rehabilitation. Hence, we neither provided any inferential analysis that could be informative to the research question.

Please explicitly state in Methods that the study was not powered or designed to detect such associations, and that the intention was to provide descriptive, hypothesis generating findings. Alternatively, consider adding limited bivariate analysis (e.g., chi square) for key outcomes, if the dataset allows

**Authors’ response:** Thank you again for the suggestions. We have explicitly mentioned about the descriptive nature of the study in lines 111 – 113:

In the quantitative part, we aimed to describe the acceptability and challenges associated with CBR, and the study design excluded any exploration of any associated factors.

As explained earlier, because of the high acceptability responses, the dataset would not allow any meaningful bivariate analysis.

Qualitative methods: more detail on rigor
The qualitative component is well conceptualized with a deductive thematic framework and rich quotes, but several methodological details are missing.
Please clarify:
Sampling strategy for key informants and FGD participants (purposive, maximum variation, criteria used).

**Authors’ response:** Thank you for the detailed suggestions on the qualitative part. We have clearly described the sampling strategy. Please check lines 132 to 139:

Four FGDs included G2D Leprosy patients and, and one FGD involved nurses with experiences in leprosy management. Participants were selected using purposive sampling to ensure inclusion of individuals with relevant knowledge and experience related to the study topic. A maximum variation approach was applied to capture diverse perspectives across different roles in sociodemographic and service contexts. Selection criteria for key informants included professional role, years of experience, and involvement in service delivery, while FGD participants were selected based on eligibility criteria such as recent experience with the service, residence in the study area, and willingness to participate.

Interview/FGD setting, language, duration, and whether audio recordings were transcribed verbatim.

**Authors’ response:** Thank you for the comment. We have detailed the process in lines 165 to 180:

Data was collected by two trained public health graduates (one female and one male) from medical and dental backgrounds. They did not have any prior relationship or interactions with the interview participants, reducing potential bias. The interviewers used probes to add clarification and to keep the discussion on topic. The KII interviews were conducted through prior appointments and at the informants’ room in their institution. The purpose of the study was explained, and permission was taken for audio recordings before the interview. Each interview took around 30 to 40 minutes to complete. Each FGD was conducted by two public health graduates simultaneously. One as the facilitator and another as the note taker. FGDs with female patients and nurses were facilitated by the female interviewer. The interview took place in a calm and quiet room in the respective hospital. Permission was taken for audio recording. Discussion lasted between 60 and 70 minutes. Interviews were conducted in Bangla using translated KII and FGD guidelines. The audio records were transcribed verbatim by the interviewers. The transcription was then translated into English by the interviewers and verified by an expert English speaker. Interviewers used interview guidelines to ensure objectivity and uniformity during the procedure.

Who conducted the interviews/FGDs (profession, sex, training, prior relationship with participants) and how reflexivity was addressed.

**Authors’ response:** Thank you for the comment. As mentioned in the above response, we have incorporated the interviewer's profession, sex, training, and prior relationship with participants in the text. The last statement mentions how we managed reflexivity by ensuring that the interviewers follow the guidelines.

How coding was done (manual vs software; how many coders; how discrepancies were resolved) and what steps were taken to ensure trustworthiness (e.g., team debriefings, triangulation).

**Authors’ response:** Thank you for the comment. We have reorganized the data analysis section into three subsections: quantitative analysis, qualitative analysis and integration of qualitative and quantitative findings. In the second subsection, we have detailed the coding and subsequent processes.

Lines 192 - 199:

FGDs were systematically coded and organized according to this framework to explore participants' perspectives on the predetermined domains of interest. Data was coded manually by two investigators (KA and AM), and any discrepancies were resolved through consultation with the third investigator (FK). To ensure trustworthiness, regular team debriefings were conducted throughout data collection and analysis to reflect on thematic conformity and minimize individual bias. Triangulation was achieved by comparing findings across different participant groups and data sources (interviews and FGDs). Field notes were maintained to support the interpretation of the data.

A brief paragraph on how the quantitative and qualitative findings were integrated (triangulated) would also strengthen the mixed methods aspect.

**Authors’ response:** Thank you for the comment. In the integration of qualitative and quantitative findings subsection, we have described the integration approach.

Lines 200 - 205:

Integration of qualitative and quantitative findings

This was undertaken at the interpretation stage using a triangulation approach. Quantitative results provided the overall patterns and magnitude of key outcomes, while qualitative data from interviews and FGDs were used to explain and contextualize these findings. Areas of convergence (agreement), complementarity (where qualitative insights added depth), and divergence (where findings differed) were systematically examined.

Inconsistencies in reporting provider reluctance
In the quantitative results, 78.37% of participants report healthcare worker reluctance to rehabilitate leprosy patients. Later in the qualitative section, the text states that “21.6% of respondents reported healthcare provider reluctance.”
This is a substantive inconsistency. Please re check the data and clarify whether these figures refer to different questions, subsamples, or are due to a reporting error. Correct the text and tables accordingly.

**Authors’ response:** Thank you for pointing this out. This was a mistake. We have removed the numbered quantitative statement from the qualitative description part.

Interpretation of extremely high acceptability
The manuscript repeatedly emphasizes “universal agreement (100%)” for many acceptability items. While this underscores demand for CBR, it also raises concerns about ceiling effects and measurement bias.
In the Discussion and Limitations, please explicitly acknowledge that:
The very high agreement may reflect how questions were framed (strongly leading toward benefits).
Social desirability bias is likely, given the stigmatizing condition and health facility setting.
The acceptability instrument may not discriminate between nuanced views.
This does not invalidate the findings but should temper the strength of some claims.

**Authors’ response:** Thank you for the comment and the overall guidance through your comments. We are grateful for your feedback. We have incorporated these limitations in the study limitations section. It now reads (lines 550 – 563):

Several limitations of the study warrant consideration. The convenience sampling approach may have introduced selection bias and limited generalizability. The calculated sample size could not be achieved, reducing the precision of the estimates. The very high levels of reported acceptability, including instances of universal agreement, should be interpreted cautiously. These findings may reflect ceiling effects, potential measurement limitations, and the framing of questions toward positive aspects of the intervention. As the interviews were conducted at the health facilities by public health graduates and because of the sensitive nature of the topic, the possibility of acquiescence and social desirability bias could not be excluded. As a result, the instrument may not have fully captured more nuanced views. The cross-sectional design precludes causal inferences about the relationship between CBR implementation and outcomes. Future longitudinal studies with larger, probability-based samples would strengthen evidence for policy decisions. The deductive thematic analysis limits the discovery of novel themes from the qualitative interviews. A phenomenological approach could be taken in further qualitative studies.

**Reviewer #2**:

The authors address an important subject of relevance to the target audience of PLOS Global Public Health. The paper’s currency is its mixed-method investigation of the acceptability and challenges of Community-Based Rehabilitation (CBR) of leprosy patients with visible (grade-2) disabilities in Bangladesh.

**Authors’ response:** Thank you for the detailed comments. We believe the comments have improved the manuscript.

It was a joy to read a well written and rigorously researched and analyzed manuscript. However, there a few minor errors that need to be addressed before it is accepted for publication:

1. Introduction section: In lines 85-87 of page 5, authors state that “Over 200,000 new leprosy cases were reported in 2016 86 (WHO, 2018) alone. In 2019, 3638 people were diagnosed as new leprosy cases. Of them, 6.94% 87 developed grade 2 disability (G2D). While this statistic is correct, nevertheless, the number of new cases quoted is 10 years old.

Action: It will be more appropriate to use more recent data e.g., new leprosy cases diagnosed in 2022, and the proportion of them that developed grade 2 disability. These data are easily available from the WHO website or from the National TB/Leprosy control program.

**Authors’ response:** Thank you for the detailed comments. We have updated the passage with the latest information and citations. Now it reads:

Lines 84 – 86:

Around 200,000 new cases of leprosy are reported every year [1]. In 2024, an estimated 172,717 incident cases of leprosy were reported worldwide[2]. Of them, 9,153 cases (5.3%) had grade 2 disability (G2D) [3].

2. Methods section: The sentence in lines 127-129 of page 7 of manuscript states: “The acceptability was assessed for CBR based on primary health centers and 128 on villages and on community-based rehabilitation in primary health care, on the possibilities of 129 creating”. This sentence has seemingly ended abruptly with the phase…on the possibilities of creating…
Action: Please crosscheck and update this sentence as it leaves the reader handing and expecting more!!!

**Authors’ response:** Thank you for the comments. The numbers “128” and “129” are actually page numbers, which might have been misplaced within the sentence during PDF conversion of the submission file. Nevertheless, based on the other reviewer’s comment, we have rewritten this part with more detail. Please check the data collection techniques subsection of the method section for the detailed changes made.

3. Study limitations and strengths: Line 488 of page 27 of the manuscript abruptly ends with …leprosy patients would…
Action: Please crosscheck and make this state complete.

**Authors’ response:** Thank you for the comments. We have base modified the section based on the feedback. Please check lines 549 to 562. It now reads:

Several limitations of the study warrant consideration. The convenience sampling approach may have introduced selection bias and limited generalizability. The calculated sample size could not be achieved, reducing the precision of the estimates. The very high levels of reported acceptability, including instances of universal agreement, should be interpreted cautiously. These findings may reflect ceiling effects, potential measurement limitations, and the framing of questions toward positive aspects of the intervention. As the interviews were conducted at the health facilities by public health graduates and because of the sensitive nature of the topic, the possibility of acquiescence and social desirability bias could not be excluded. As a result, the instrument may not have fully captured more nuanced views. The cross-sectional design precludes causal inferences about the relationship between CBR implementation and outcomes. Future longitudinal studies with larger, probability-based samples would strengthen evidence for policy decisions. The deductive thematic analysis limits the discovery of novel themes from the qualitative interviews. A phenomenological approach could be taken in further qualitative studies.
