# Supplementary material for: Acceptability and challenges of community-based rehabilitation (CBR) of leprosy patients with grade-2 disability in Bangladesh
Source: PLOS Glob Public Health. 2026 Aug 7;6(8):e0006994. doi: 10.1371/journal.pgph.0006994 (PMC13450763; doi:10.1371/journal.pgph.0006994)
Supplement: Peer review history

# Author comment: Author response to Decision Letter 0

**DOI:** 10.1371/journal.pgph.0006994.r001

**Transfer Alert**

This paper was transferred from another journal. As a result, its full editorial history (including decision letters, peer reviews and author responses) may not be present.

29 Dec 2025

# Peer review: Decision Letter 0

**DOI:** 10.1371/journal.pgph.0006994.r002

**Authors:** Alejandro Torrado Pacheco

18 Feb 2026

PGPH-D-25-04014

Acceptability and Challenges of Community-Based Rehabilitation (CBR) of Leprosy Patients with Grade-2 Disability in Bangladesh

PLOS Global Public Health

Dear Dr. Akhtar,

Thank you for submitting your manuscript to PLOS Global Public Health. After careful consideration, we feel that it has merit but does not fully meet PLOS Global Public Health’s publication criteria as it currently stands. Therefore, we invite you to submit a revised version of the manuscript that addresses the points raised during the review process.

The manuscript has been evaluated by two reviewers, and their comments are available below.

The reviewers have raised a number of major concerns. They feel the manuscript requires substantial revisions to clarify the methods used, as well as other aspects such as the sample size calculation.

Could you please carefully revise the manuscript to address all comments raised?

Please submit your revised manuscript by Apr 04 2026 11:59PM. If you will need more time than this to complete your revisions, please reply to this message or contact the journal office at globalpubhealth@plos.org. When you're ready to submit your revision, log on to https://www.editorialmanager.com/pgph/ and select the 'Submissions Needing Revision' folder to locate your manuscript file.

Please include the following items when submitting your revised manuscript:

- A letter that responds to each point raised by the editor and reviewer(s). You should upload this letter as a separate file labeled 'Response to Reviewers'.
- A marked-up copy of your manuscript that highlights changes made to the original version. You should upload this as a separate file labeled 'Revised Manuscript with Track Changes'.
- An unmarked version of your revised paper without tracked changes. You should upload this as a separate file labeled 'Manuscript'.

Guidelines for resubmitting your figure files are available below the reviewer comments at the end of this letter.

We look forward to receiving your revised manuscript.

Kind regards,

Alejandro Torrado Pacheco, PhD

PLOS Editor

PLOS Global Public Health

Journal Requirements:

1. Please amend your detailed Financial Disclosure statement. This is published with the article. It must therefore be completed in full sentences and contain the exact wording you wish to be published.

i. Please clarify all sources of financial support for your study. List the grants, grant numbers, and organizations that funded your study, including funding received from your institution. Please note that suppliers of material support, including research materials, should be recognized in the Acknowledgements section rather than in the Financial Disclosure.

ii. State the initials, alongside each funding source, of each author to receive each grant. For example: "This work was supported by the National Institutes of Health (####### to AM; ###### to CJ) and the National Science Foundation (###### to AM)."

iii. State what role the funders took in the study. If the funders had no role in your study, please state: “The funders had no role in study design, data collection and analysis, decision to publish, or preparation of the manuscript.”

iv. If any authors received a salary from any of your funders, please state which authors and which funders.

2. Please upload separate figure files in .tif or .eps format. Also, remove the figures from your manuscript file but keep the legends.

For more information about figure files please see our guidelines:

https://journals.plos.org/globalpublichealth/s/figures

https://journals.plos.org/globalpublichealth/s/figures#loc-file-requirements

3. We have noticed that you have uploaded Supporting Information files, but you have not included a list of legends. Please add a full list of legends for your Supporting Information files after the references list.

4. Please ensure that your Ethics Statement is available in its entirety at the beginning of your Methods section, under a subheading 'Ethics Statement'.

5. In the online submission form, you indicated that “De-identified data is available upon reasonable request from the corresponding author.”.

All PLOS journals now require all data underlying the findings described in their manuscript to be freely available to other researchers, either

1. In a public repository,

2. Within the manuscript itself, or

3. Uploaded as supplementary information.

This policy applies to all data except where public deposition would breach compliance with the protocol approved by your research ethics board. If your data cannot be made publicly available for ethical or legal reasons (e.g., public availability would compromise patient privacy), please explain your reasons by return email and your exemption request will be escalated to the editor for approval. Your exemption request will be handled independently and will not hold up the peer review process, but will need to be resolved should your manuscript be accepted for publication. One of the Editorial team will then be in touch if there are any issues.

If the reviewer comments include a recommendation to cite specific previously published works, please review and evaluate these publications to determine whether they are relevant and should be cited. There is no requirement to cite these works unless the editor has indicated otherwise.

Additional Editor Comments (if provided):

[Note: HTML markup is below. Please do not edit.]

Reviewers' comments:

Reviewer's Responses to Questions

**Comments to the Author**

1. Does this manuscript meet PLOS Global Public Health’s publication criteria? Is the manuscript technically sound, and do the data support the conclusions? The manuscript must describe methodologically and ethically rigorous research with conclusions that are appropriately drawn based on the data presented.

Reviewer #1: Partly

Reviewer #2: Yes

**********

2. Has the statistical analysis been performed appropriately and rigorously?

Reviewer #1: Yes

Reviewer #2: Yes

**********

3. Have the authors made all data underlying the findings in their manuscript fully available (please refer to the Data Availability Statement at the start of the manuscript PDF file)?

The PLOS Data policy requires authors to make all data underlying the findings described in their manuscript fully available without restriction, with rare exception. The data should be provided as part of the manuscript or its supporting information, or deposited to a public repository. For example, in addition to summary statistics, the data points behind means, medians and variance measures should be available. If there are restrictions on publicly sharing data—e.g. participant privacy or use of data from a third party—those must be specified.

Reviewer #1: Yes

Reviewer #2: Yes

**********

4. Is the manuscript presented in an intelligible fashion and written in standard English?

PLOS Global Public Health does not copyedit accepted manuscripts, so the language in submitted articles must be clear, correct, and unambiguous. Any typographical or grammatical errors should be corrected at revision, so please note any specific errors here.

Reviewer #1: Yes

Reviewer #2: Yes

**********

5. Review Comments to the Author

Please use the space provided to explain your answers to the questions above. You may also include additional comments for the author, including concerns about dual publication, research ethics, or publication ethics. (Please upload your review as an attachment if it exceeds 20,000 characters)

Reviewer #1: This is an important and timely mixed methods study assessing the acceptability and perceived challenges of community based rehabilitation (CBR) for people affected by leprosy with grade 2 disability (G2D) in Bangladesh. The work adds valuable empirical evidence from both patients and providers in a high burden setting. However, several methodological clarifications, interpretive refinements, and minor inconsistencies need to be addressed before the paper is suitable for publication.

Sampling strategy and generalizability

The quantitative component uses convenience sampling of G2D patients from selected government and NGO hospitals in three divisions. This is appropriate for a hard to reach population but limits generalizability.

Please describe the inclusion and exclusion criteria explicitly (e.g., age threshold, current treatment status, cognitive/communication ability). Clarify how patients were approached and whether any refused participation

Explain why these three divisions and these specific facilities were chosen (e.g., higher leprosy burden, presence of specialized services.

The sample size calculation (n = Z^2 pq/d^2) assumed 50% prevalence of acceptability but the achieved sample was 356 instead of 384. Please (a) write out the full formula clearly, (b) explain the practical reasons for stopping at 356, and (c) briefly comment on the impact on precision.

Measurement of “acceptability” and “challenges”

The section describing how acceptability and challenges were measured is currently brief and partially unclear (e.g., “The acceptability was assessed for CBR based on primary health centers and on villages…”).

Please clearly specify:

The response options (e.g., Likert scale vs yes/no) for each set of items

How items were developed (based on literature, expert consultation, pre testing) and whether any reliability assessment was done (even simple internal consistency).

Whether items were asked in a standard order and if interviewers had probes or showcards.

Given the very high levels of agreement (many items at 100%), please consider and discuss the possibility of acquiescence and social desirability bias, particularly as interviews were conducted in health facilities by health trained data collectors

Descriptive focus and lack of inferential analysis

The quantitative analysis is purely descriptive, which is acceptable for an exploratory study, but why were no associations were examined (e.g., acceptability by sex, age, education, type of disability).

Please explicitly state in Methods that the study was not powered or designed to detect such associations, and that the intention was to provide descriptive, hypothesis generating findings. Alternatively, consider adding limited bivariate analysis (e.g., chi square) for key outcomes, if the dataset allows

Qualitative methods: more detail on rigor

The qualitative component is well conceptualized with a deductive thematic framework and rich quotes, but several methodological details are missing.

Please clarify:

Sampling strategy for key informants and FGD participants (purposive, maximum variation, criteria used).

Interview/FGD setting, language, duration, and whether audio recordings were transcribed verbatim.

Who conducted the interviews/FGDs (profession, sex, training, prior relationship with participants) and how reflexivity was addressed.

How coding was done (manual vs software; how many coders; how discrepancies were resolved) and what steps were taken to ensure trustworthiness (e.g., team debriefings, triangulation).

A brief paragraph on how the quantitative and qualitative findings were integrated (triangulated) would also strengthen the mixed methods aspect.

Inconsistencies in reporting provider reluctance

In the quantitative results, 78.37% of participants report healthcare worker reluctance to rehabilitate leprosy patients. Later in the qualitative section, the text states that “21.6% of respondents reported healthcare provider reluctance.”

This is a substantive inconsistency. Please re check the data and clarify whether these figures refer to different questions, subsamples, or are due to a reporting error. Correct the text and tables accordingly.

Interpretation of extremely high acceptability

The manuscript repeatedly emphasizes “universal agreement (100%)” for many acceptability items. While this underscores demand for CBR, it also raises concerns about ceiling effects and measurement bias.

In the Discussion and Limitations, please explicitly acknowledge that:

The very high agreement may reflect how questions were framed (strongly leading toward benefits).

Social desirability bias is likely, given the stigmatizing condition and health facility setting.

The acceptability instrument may not discriminate between nuanced views.

This does not invalidate the findings but should temper the strength of some claims.

Reviewer #2: The authors address an important subject of relevance to the target audience of PLOS Global Public Health. The paper’s currency is its mixed-method investigation of the acceptability and challenges of Community-Based Rehabilitation (CBR) of leprosy patients with visible (grade-2) disabilities in Bangladesh.

It was a joy to read a well written and rigorously researched and analyzed manuscript However, there a few minor errors that need to be addressed before it is accepted for publication:

1. Introduction section: In lines 85-87 of page 5, authors state that “Over 200,000 new leprosy cases were reported in 2016 86 (WHO, 2018) alone. In 2019, 3638 people were diagnosed as new leprosy cases. Of them, 6.94% 87 developed grade 2 disability (G2D). While this statistic is correct, nevertheless, the number of new cases quoted is 10 years old.

Action: It will be more appropriate to use more recent data e.g., new leprosy cases diagnosed in 2022, and the proportion of them that developed grade 2 disability. These data are easily available from the WHO website or from the National TB/Leprosy control program.

2. Methods section: The sentence in lines 127-129 of page 7 of manuscript states: “The acceptability was assessed for CBR based on primary health centers and 128 on villages and on community-based rehabilitation in primary health care, on the possibilities of 129 creating”. This sentence has seemingly ended abruptly with the phase…on the possibilities of creating…

Action: Please crosscheck and update this sentence as it leaves the reader handing and expecting more!!!

3. Study limitations and strengths: Line 488 of page 27 of the manuscript abruptly ends with …leprosy patients would…

Action: Please crosscheck and make this state complete.

**********

6. PLOS authors have the option to publish the peer review history of their article (what does this mean?). If published, this will include your full peer review and any attached files.

**Do you want your identity to be public for this peer review?**  If you choose “no”, your identity will remain anonymous but your review may still be made public.

For information about this choice, including consent withdrawal, please see our Privacy Policy.

Reviewer #1: **Yes:**  Dr.Visweswara Rao Guthi

Reviewer #2: **Yes:**  Bassey Ebenso

**********

[NOTE: If reviewer comments were submitted as an attachment file, they will be attached to this email and accessible via the submission site. Please log into your account, locate the manuscript record, and check for the action link "View Attachments". If this link does not appear, there are no attachment files.]

-->Figure Resubmissions:

While revising your submission, we strongly recommend that you use PLOS’s NAAS tool (https://ngplosjournals.pagemajik.ai/artanalysis) to test your figure files. NAAS can convert your figure files to the TIFF file type and meet basic requirements (such as print size, resolution), or provide you with a report on issues that do not meet our requirements and that NAAS cannot fix.

After uploading your figures to PLOS’s NAAS tool - https://ngplosjournals.pagemajik.ai/artanalysis, NAAS will process the files provided and display the results in the "Uploaded Files" section of the page as the processing is complete. If the uploaded figures meet our requirements (or NAAS is able to fix the files to meet our requirements), the figure will be marked as "fixed" above. If NAAS is unable to fix the files, a red "failed" label will appear above. When NAAS has confirmed that the figure files meet our requirements, please download the file via the download option, and include these NAAS processed figure files when submitting your revised manuscript.

# Author comment: Author response to Decision Letter 1

**DOI:** 10.1371/journal.pgph.0006994.r003

15 Apr 2026

Attachment Submitted filename: Response to reviewers.docx

# Peer review: Decision Letter 1

**DOI:** 10.1371/journal.pgph.0006994.r004

**Authors:** Helen Howard

11 May 2026

PGPH-D-25-04014R1

Acceptability and Challenges of Community-Based Rehabilitation (CBR) of Leprosy Patients with Grade-2 Disability in Bangladesh

PLOS Global Public Health

Dear Dr. Akhtar,

Thank you for submitting your manuscript to PLOS Global Public Health. After careful consideration, we feel that it has merit but does not fully meet PLOS Global Public Health’s publication criteria as it currently stands. Therefore, we invite you to submit a revised version of the manuscript that addresses the points raised during the review process.

Please submit your revised manuscript by Jul 07 2026 11:59PM. If you will need more time than this to complete your revisions, please reply to this message or contact the journal office at globalpubhealth@plos.org. When you're ready to submit your revision, log on to https://www.editorialmanager.com/pgph/ and select the 'Submissions Needing Revision' folder to locate your manuscript file.

Please include the following items when submitting your revised manuscript:

- A letter that responds to each point raised by the editor and reviewer(s). You should upload this letter as a separate file labeled 'Response to Reviewers'.
- A marked-up copy of your manuscript that highlights changes made to the original version. You should upload this as a separate file labeled 'Revised Manuscript with Track Changes'.
- An unmarked version of your revised paper without tracked changes. You should upload this as a separate file labeled 'Manuscript'.

Guidelines for resubmitting your figure files are available below the reviewer comments at the end of this letter.

As the corresponding author, your ORCID iD is verified in the submission system and will appear in the published article. PLOS supports the use of ORCID, and we encourage all coauthors to register for an ORCID iD and use it as well. Please encourage your coauthors to verify their ORCID iD within the submission system before final acceptance, as unverified ORCID iDs will not appear in the published article. *Only*  the individual author can complete the verification step; PLOS staff *cannot*  verify ORCID iDs on behalf of authors.

We look forward to receiving your revised manuscript.

Kind regards,

Helen Howard

Staff Editor

PLOS Global Public Health

Journal Requirements:

If the reviewer comments include a recommendation to cite specific previously published works, please review and evaluate these publications to determine whether they are relevant and should be cited. There is no requirement to cite these works unless the editor has indicated otherwise.

Additional Editor Comments (if provided):

[Note: HTML markup is below. Please do not edit.]

Reviewers' comments:

Reviewer's Responses to Questions

**Comments to the Author**

1. If the authors have adequately addressed your comments raised in a previous round of review and you feel that this manuscript is now acceptable for publication, you may indicate that here to bypass the “Comments to the Author” section, enter your conflict of interest statement in the “Confidential to Editor” section, and submit your "Accept" recommendation.

Reviewer #1: All comments have been addressed

Reviewer #2: All comments have been addressed

**********

2. Does this manuscript meet PLOS Global Public Health’s publication criteria? Is the manuscript technically sound, and do the data support the conclusions? The manuscript must describe methodologically and ethically rigorous research with conclusions that are appropriately drawn based on the data presented.

Reviewer #1: Yes

Reviewer #2: Yes

**********

3. Has the statistical analysis been performed appropriately and rigorously?

Reviewer #1: Yes

Reviewer #2: Yes

**********

4. Have the authors made all data underlying the findings in their manuscript fully available (please refer to the Data Availability Statement at the start of the manuscript PDF file)?

The PLOS Data policy requires authors to make all data underlying the findings described in their manuscript fully available without restriction, with rare exception. The data should be provided as part of the manuscript or its supporting information, or deposited to a public repository. For example, in addition to summary statistics, the data points behind means, medians and variance measures should be available. If there are restrictions on publicly sharing data—e.g. participant privacy or use of data from a third party—those must be specified.

Reviewer #1: Yes

Reviewer #2: Yes

**********

5. Is the manuscript presented in an intelligible fashion and written in standard English?

PLOS Global Public Health does not copyedit accepted manuscripts, so the language in submitted articles must be clear, correct, and unambiguous. Any typographical or grammatical errors should be corrected at revision, so please note any specific errors here.

Reviewer #1: Yes

Reviewer #2: Yes

**********

6. Review Comments to the Author

Please use the space provided to explain your answers to the questions above. You may also include additional comments for the author, including concerns about dual publication, research ethics, or publication ethics. (Please upload your review as an attachment if it exceeds 20,000 characters)

Reviewer #1: The mixed-methods design yields robust evidence of high acceptability (often 100%) alongside practical barriers, aligning well with PLOS Global Public Health's scope on global health equity

Universal agreement on acceptability items risks ceiling effects; while acknowledged, consider validating the yes/no questionnaire in future work or adding Likert scales for nuance. Sample size fell short (356/384) due to time constraints—power implications are noted, but report confidence intervals around key proportions .

In methods section Required clarifications:

Inclusion and exclusion criteria need to define clearly

Sample size justification (power calculation?)

Response rate

In qualitative part, add information regarding data saturation for KII and FGDs.

In results summary of qualitatative part can be presented for better readability.

Limitations shoud be explained futher regarding Convenience sampling bias, Hospital-based recruitment bias, Social desirability bias, Cross-sectional design limitations, Generalizability issues

Reviewer #2: The authors have sufficiently addressed my comments.

**********

7. PLOS authors have the option to publish the peer review history of their article (what does this mean?). If published, this will include your full peer review and any attached files.

**Do you want your identity to be public for this peer review?**  If you choose “no”, your identity will remain anonymous but your review may still be made public.

For information about this choice, including consent withdrawal, please see our Privacy Policy.

Reviewer #1: **Yes:**  Dr.Visweswara Rao Guthi

Reviewer #2: **Yes:**  Dr. Bassey Ebenso

**********

[NOTE: If reviewer comments were submitted as an attachment file, they will be attached to this email and accessible via the submission site. Please log into your account, locate the manuscript record, and check for the action link "View Attachments". If this link does not appear, there are no attachment files.]

-->Figure Resubmissions:

While revising your submission, we strongly recommend that you use PLOS’s NAAS tool (https://ngplosjournals.pagemajik.ai/artanalysis) to test your figure files. NAAS can convert your figure files to the TIFF file type and meet basic requirements (such as print size, resolution), or provide you with a report on issues that do not meet our requirements and that NAAS cannot fix.

After uploading your figures to PLOS’s NAAS tool - https://ngplosjournals.pagemajik.ai/artanalysis, NAAS will process the files provided and display the results in the "Uploaded Files" section of the page as the processing is complete. If the uploaded figures meet our requirements (or NAAS is able to fix the files to meet our requirements), the figure will be marked as "fixed" above. If NAAS is unable to fix the files, a red "failed" label will appear above. When NAAS has confirmed that the figure files meet our requirements, please download the file via the download option, and include these NAAS processed figure files when submitting your revised manuscript.

Attachment Submitted filename: PGPH-D-25-04014_R1.pdf

# Author comment: Author response to Decision Letter 2

**DOI:** 10.1371/journal.pgph.0006994.r005

27 Jun 2026

Attachment Submitted filename: Response_to_reviewers_auresp_2.docx

# Peer review: Decision Letter 2

**DOI:** 10.1371/journal.pgph.0006994.r006

**Authors:** Julia Robinson

17 Jul 2026

Acceptability and Challenges of Community-Based Rehabilitation (CBR) of Leprosy Patients with Grade-2 Disability in Bangladesh

PGPH-D-25-04014R2

Dear Associate Professor Akhtar,

We are pleased to inform you that your manuscript 'Acceptability and Challenges of Community-Based Rehabilitation (CBR) of Leprosy Patients with Grade-2 Disability in Bangladesh' has been provisionally accepted for publication in PLOS Global Public Health.

Before your manuscript can be formally accepted you will need to complete some formatting changes, which you will receive in a follow up email. A member of our team will be in touch with a set of requests.

Please note that your manuscript will not be scheduled for publication until you have made the required changes, so a swift response is appreciated.

IMPORTANT: The editorial review process is now complete. PLOS will only permit corrections to spelling, formatting or significant scientific errors from this point onwards. Requests for major changes, or any which affect the scientific understanding of your work, will cause delays to the publication date of your manuscript.

If your institution or institutions have a press office, please notify them about your upcoming paper to help maximize its impact. If they'll be preparing press materials, please inform our press team as soon as possible -- no later than 48 hours after receiving the formal acceptance. Your manuscript will remain under strict press embargo until 2 pm Eastern Time on the date of publication. For more information, please contact globalpubhealth@plos.org.

Thank you again for supporting Open Access publishing; we are looking forward to publishing your work in PLOS Global Public Health.

Best regards,

Julia Robinson

Executive Editor

PLOS Global Public Health

***********************************************************

Reviewer Comments (if any, and for reference):

Reviewer's Responses to Questions

**Comments to the Author**

1. If the authors have adequately addressed your comments raised in a previous round of review and you feel that this manuscript is now acceptable for publication, you may indicate that here to bypass the “Comments to the Author” section, enter your conflict of interest statement in the “Confidential to Editor” section, and submit your "Accept" recommendation.

Reviewer #1: All comments have been addressed

**********

2. Does this manuscript meet PLOS Global Public Health’s publication criteria? Is the manuscript technically sound, and do the data support the conclusions? The manuscript must describe methodologically and ethically rigorous research with conclusions that are appropriately drawn based on the data presented.

Reviewer #1: Yes

**********

3. Has the statistical analysis been performed appropriately and rigorously?

Reviewer #1: Yes

**********

4. Have the authors made all data underlying the findings in their manuscript fully available (please refer to the Data Availability Statement at the start of the manuscript PDF file)?

The PLOS Data policy requires authors to make all data underlying the findings described in their manuscript fully available without restriction, with rare exception. The data should be provided as part of the manuscript or its supporting information, or deposited to a public repository. For example, in addition to summary statistics, the data points behind means, medians and variance measures should be available. If there are restrictions on publicly sharing data—e.g. participant privacy or use of data from a third party—those must be specified.

Reviewer #1: Yes

**********

5. Is the manuscript presented in an intelligible fashion and written in standard English?

PLOS Global Public Health does not copyedit accepted manuscripts, so the language in submitted articles must be clear, correct, and unambiguous. Any typographical or grammatical errors should be corrected at revision, so please note any specific errors here.

Reviewer #1: Yes

**********

6. Review Comments to the Author

Please use the space provided to explain your answers to the questions above. You may also include additional comments for the author, including concerns about dual publication, research ethics, or publication ethics. (Please upload your review as an attachment if it exceeds 20,000 characters)

Reviewer #1: Authors have answers all the comments

**********

7. PLOS authors have the option to publish the peer review history of their article (what does this mean?). If published, this will include your full peer review and any attached files.

**Do you want your identity to be public for this peer review?**  If you choose “no”, your identity will remain anonymous but your review may still be made public.

For information about this choice, including consent withdrawal, please see our Privacy Policy.

Reviewer #1: **Yes:**  Dr.Visweswara Rao Guthi

**********
